# Deep learning reveals antimicrobial peptides within prions

**DOI:** 10.1038/s41564-026-02408-1

**Published:** 2026-06-19

**Authors:** Marcelo D. T. Torres, Fangping Wan, Cesar de la Fuente-Nunez

**Affiliations:** 1 Machine Biology Group, Departments of Psychiatry and Microbiology, Institute for Biomedical Informatics, Institute for Translational Medicine and Therapeutics, Perelman School of Medicine, University of Pennsylvania, Philadelphia, PA USA; 2 Departments of Bioengineering and Chemical and Biomolecular Engineering, School of Engineering and Applied Science, University of Pennsylvania, Philadelphia, PA USA; 3 Department of Chemistry, School of Arts and Sciences, University of Pennsylvania, Philadelphia, PA USA; 4 Penn Institute for Computational Science, University of Pennsylvania, Philadelphia, PA USA

**Keywords:** Microbiology, Antimicrobials

## Abstract

Prion and prion-like proteins are classically associated with protein misfolding, but amyloidogenic sequences can also participate in host defence. Here, using deep learning, we screened 19.3 million fragments from 2,897 curated prion-related proteins and identified 1,179 candidate antimicrobial peptides, which we term prionins. Among 75 synthesized prionins, 59 inhibited bacterial pathogens, 53 perturbed membranes and 2 reduced *Acinetobacter baumannii* infection burden in mice.

## Abstract (web summary)

Deep learning reveals that prion-related proteins contain hidden ‘prionin’ peptides with antibacterial activity, including two candidates that reduced infections in mice.

## Main

Prions are best known for templated conformational conversion and neurodegeneration, yet amyloidogenic proteins are increasingly viewed as functionally diverse rather than uniformly pathological. In parallel, antimicrobial peptides (AMPs) have emerged as central effectors of host defence and as promising templates for antibiotic discovery against drug-resistant pathogens^1,2^. Several amyloid-associated proteins, including amyloid-β and the cellular prion protein, have been reported to display antimicrobial or host-protective activities^3–9^, raising the possibility that aggregation-prone proteins may encode cryptic antimicrobial fragments within their primary sequence. Whether such encrypted peptides are broadly embedded across prion and prion-like proteins has not been systematically examined.

**Fig. 1:**
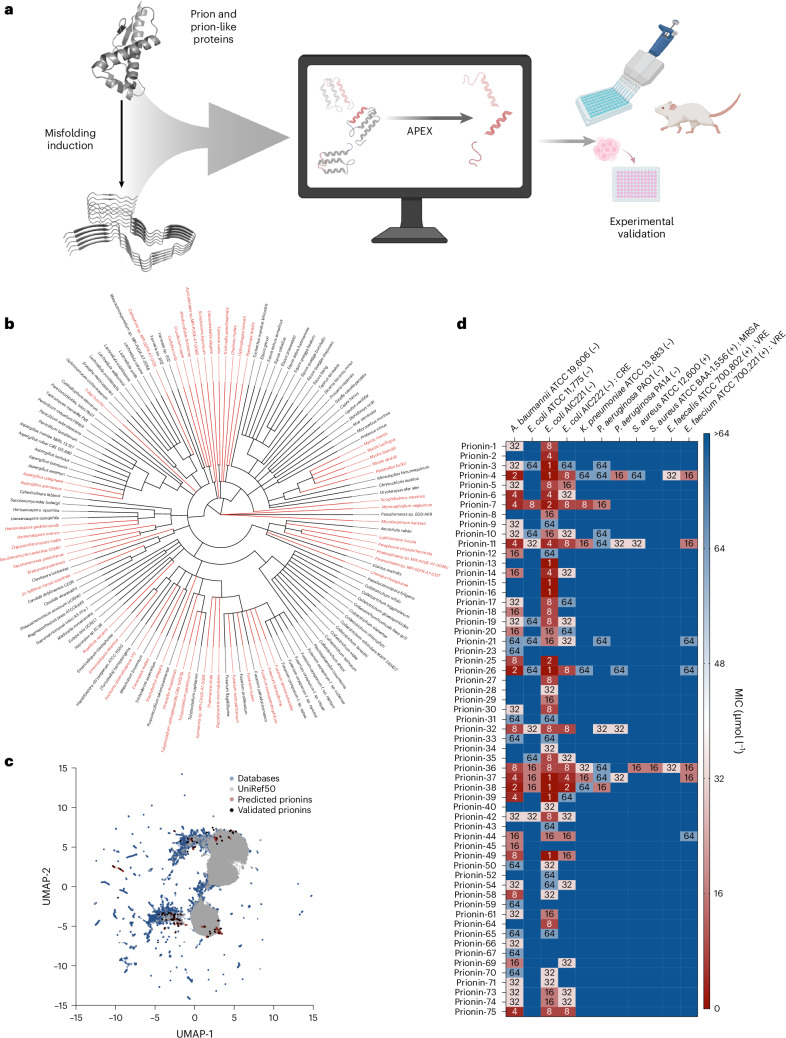
Deep learning discovery and validation of prionins. **a**, A schematic of the discovery workflow. Prion and prion-like proteins curated from UniProt were fragmented into all unique 8–50-residue peptides and screened with APEX 1.1. **b**, The taxonomic distribution of source organisms contributing candidate prionins. **c**, UMAP visualization of sequence space comparing candidate prionins with peptides from the in-house APEX dataset and curated AMP databases. **d**, A heat map of MIC values for the 75 synthesized prionins tested against 11 clinically relevant pathogens, including resistant strains. Peptides were tested from 1 to 64 μmol l^−1^; values shown are modal MICs from three biological replicates. CRE, colistin-resistant *Escherichia coli*; VRE, vancomycin-resistant *Enterococci*. Illustrations in **a** created in BioRender; De La Fuente-Nunez, C. https://biorender.com/b7rv7pg (2026). Source data

**Extended Data Fig. 1:**
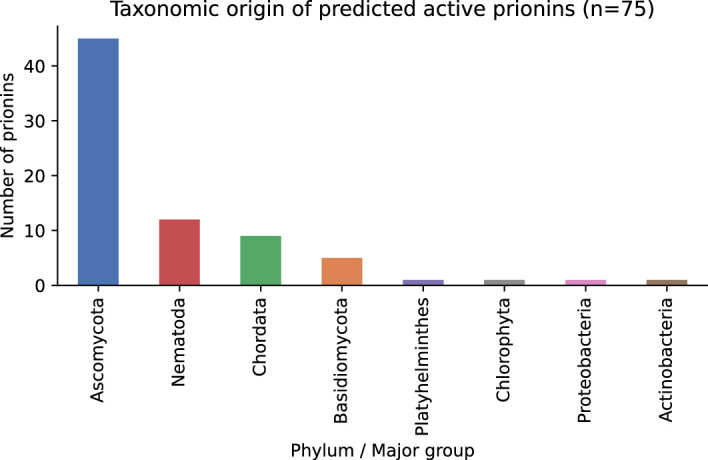
Taxonomic origin of the 75 predicted active prionins. Bars show peptide counts per phylum. Source data

We tested this hypothesis by mining prion-related proteins with APEX 1.1, a deep learning framework for AMP discovery^10–12^. We curated 2,897 UniProt entries annotated as prion or prion-related and enumerated all unique 8–50-residue fragments, yielding 19,324,138 candidate peptides. Across this space, APEX identified 1,179 fragments with a predicted median MIC of ≤64 μmol l^−1^ across an 11-pathogen panel (*Acinetobacter baumannii* ATCC 19606, *Escherichia coli* ATCC 11775, *Escherichia coli* AIC221, *Escherichia coli* AIC222, *Klebsiella pneumoniae* ATCC 13883, *Pseudomonas aeruginosa* PAO1, *Pseudomonas aeruginosa* PA14, *Staphylococcus aureus* ATCC 12600, *Staphylococcus aureus* ATCC BAA^−^1556, *Enterococcus faecalis* ATCC 700802 and *Enterococcus faecium* ATCC 700221). We term these peptides prionins. Candidate prionins arose from 1,068 organisms spanning fungi, metazoans and unicellular eukaryotes (Extended Data Fig. 1), indicating that encrypted antimicrobial potential is distributed broadly across prion-associated sequence space (Fig. 1a,b).

**Extended Data Fig. 2:**
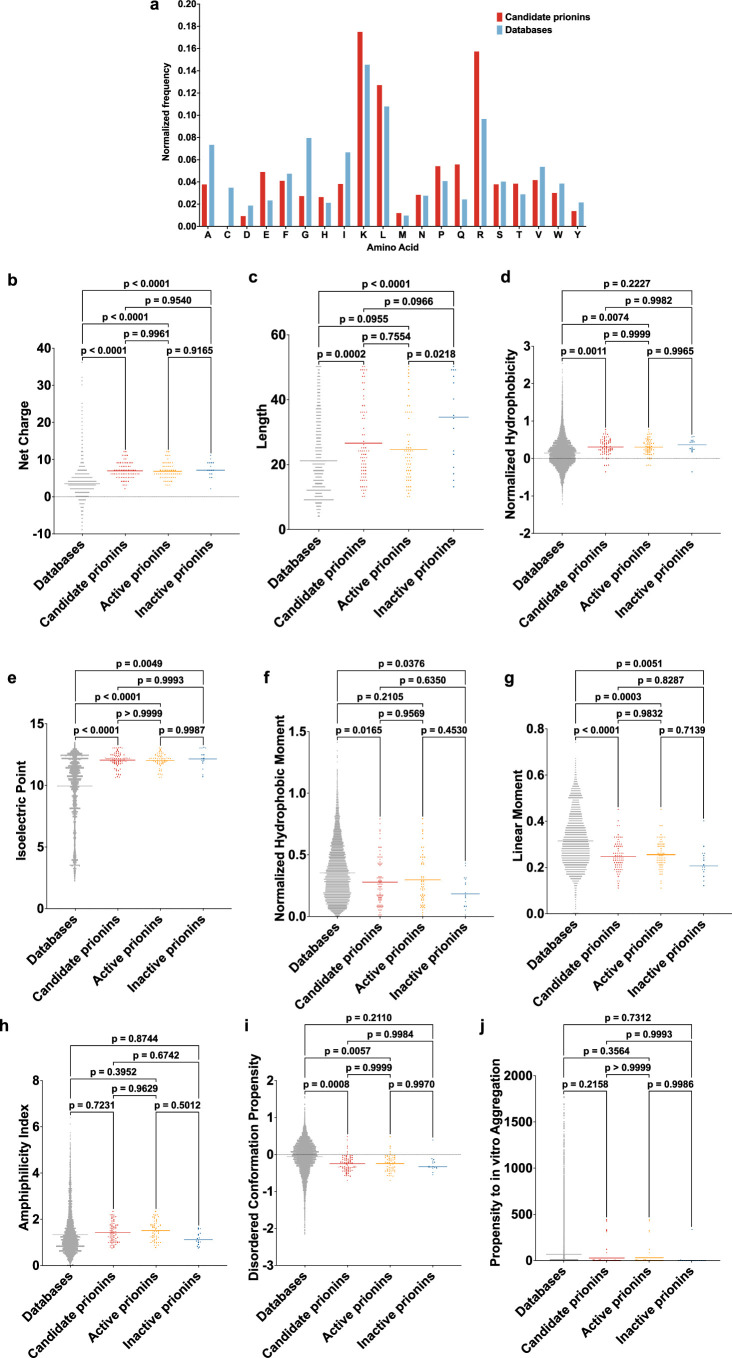
Physicochemical features of prionins compared to AMPs from databases. **(a)** Comparison of amino acid frequency in prionins with known AMPs from the DBAASP, APD3, and DRAMP 3.0 databases. Distribution of some of the most important physicochemical properties for peptides with predicted antimicrobial activity, compared with AMPs from DBAASP, APD3, and DRAMP 3.0: **(b)** net charge, **(c)** length, **(d)** and normalized hydrophobicity. **(e)** Isoelectric point, **(f)** normalized hydrophobic moment, and **(g)** linear moment, reflecting the amphipathicity of the molecules, which directly influences their interactions with bacterial membranes. **(h)** Amphiphilicity index and **(i)** disordered conformation propensity, both of which are closely correlated with the mechanism of action, specifically how peptides interact with membrane lipids to exert antimicrobial activity. **(j)** Propensity to aggregate *in vitro*, correlated with the supramolecular arrangement of the molecules and potential toxicity. Statistical significance in **b-j** was determined using two-tailed t-tests followed by Mann–Whitney test; p-values < 0.0001. The solid line in **b-j** represents the mean value for each group. Source data

To place prionins in the context of known antimicrobial repertoires, we compared them with peptides from our in-house training set and curated AMPs from the Database of Antimicrobial Activity and Structure of Peptides (DBAASP)^13^, the Antimicrobial Peptide Database (Version 3) (APD3)^14^ and the Data Repository of Antimicrobial Peptides (DRAMP)^15^. Sequence-space projection showed that prionins overlap only partially with known AMPs, consistent with both convergent and unexplored sequences (Fig. 1c). Relative to database AMPs, candidate prionins were, on average, longer, more cationic and more hydrophobic, while not uniformly maximizing classical amphipathic patterning (Extended Data Fig. 2). Thus, prion-derived fragments occupy a related but distinguishable physicochemical landscape compatible with membrane-targeting activity (Extended Data Fig. 2).

We next selected 75 top-ranked, sequence-diverse peptides for synthesis, enforcing <70% similarity to known AMPs and among selected candidates themselves. These 75 peptides originated from phylogenetically diverse proteins, including canonical prion proteins, prion-like Q/N-rich proteins and prion-inhibition proteins. Experimental testing against clinically relevant pathogens showed that 59 of 75 prionins inhibited at least one strain at ≤64 μmol l^−1^, including multidrug-resistant isolates (Fig. 1d). Forty-two peptides displayed minimum inhibitory concentrations (MICs) of ≤16 μmol l^−1^ against at least one pathogen. Activity was concentrated against Gram-negative bacteria, whereas only a minority of prionins inhibited Gram-positive organisms under the tested conditions. These data establish a high experimental hit rate for prioritized peptides derived from prion-related proteins.

**Fig. 2:**
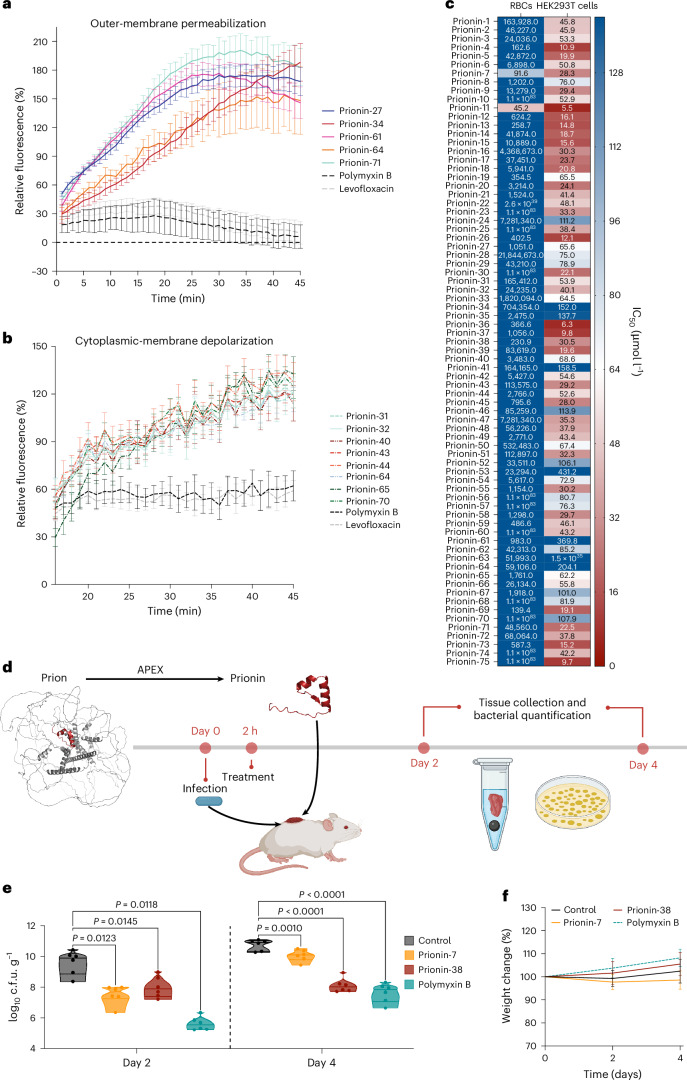
Mechanism, selectivity, and in vivo efficacy of prionins. **a**, Outer-membrane permeabilization of *E. coli* AIC221 measured by NPN uptake for representative active prionins tested at their MIC values. **b**, Cytoplasmic-membrane depolarization measured by DiSC_3_-5 for representative active prionins. The initial time reported is 15 min, after the stabilization of the probe in the membrane, when the peptide was added. **c**, A comparison of antimicrobial activity with haemolytic (HC_50_) and cytotoxic (CC_50_) measurements identifies a subset of selective prionins. Experiments reported in **a**, **b** and **c** were performed in three independent replicates. **d**, A schematic representation of the skin abscess mouse model used to assess the anti-infective activity of prionins (*n* = 6) against *A. baumannii* ATCC 19606. **e**, Bacterial burden in a murine skin-abscess model of *A. baumannii* infection following topical treatment with prionin-7, prionin-38 or polymyxin B. **f**, Mouse body weight over the course of treatment. The limit of detection for the c.f.u. quantification is log_10_ c.f.u. is 2. In **e**, the points represent individual mice (*n* = 6 per group); statistical significance was determined by one-way ANOVA with Dunnett’s test; *P* values are shown in the graphs. Bacterial burdens are shown as violin plots with individual data points overlaid; each point represents one animal. The horizontal line within each violin indicates the mean and violin width reflects the distribution of values within each group. Statistical comparisons were performed separately for each time point using ordinary one-way ANOVA followed by Dunnett’s two-sided multiple comparisons test versus the control group. Exact *P* values are shown above the indicated comparisons. In **a**, **b**, and **f**, error bars indicate standard deviation and lines indicate mean values. Schematic in **d** created in BioRender; De La Fuente-Nunez, C. https://biorender.com/95zyip2 (2026). Source data

**Extended Data Fig. 3:**
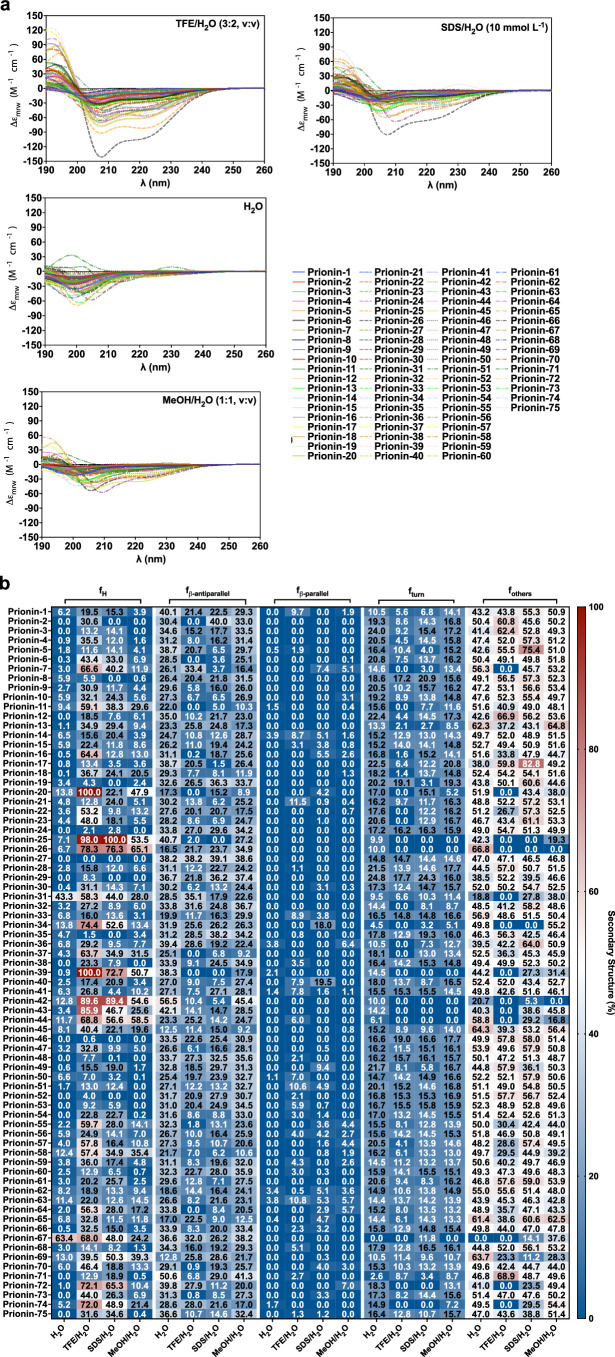
Circular dichroism spectra of prionins. Circular dichroism experiments were conducted with prionins using a J-1500 Jasco circular dichroism spectrophotometer. **(a)** The spectra were recorded in four different media: water, 60% trifluoroethanol in water, 50% methanol in water, and sodium dodecyl sulfate (SDS) in water (10 mmol L^−1^), after three accumulations at 25 °C, using a 1 mm path length quartz cell, between 260 and 190 nm at 50 nm min^−1^, with a bandwidth of 0.5 nm. The concentration of all peptides tested was 50 μmol L^−1^. **(b)** Heatmap with the percentage of secondary structure found for each peptide in the four different solvents. Secondary structure fraction was calculated using the BeStSel server. Source data

**Extended Data Fig. 4:**
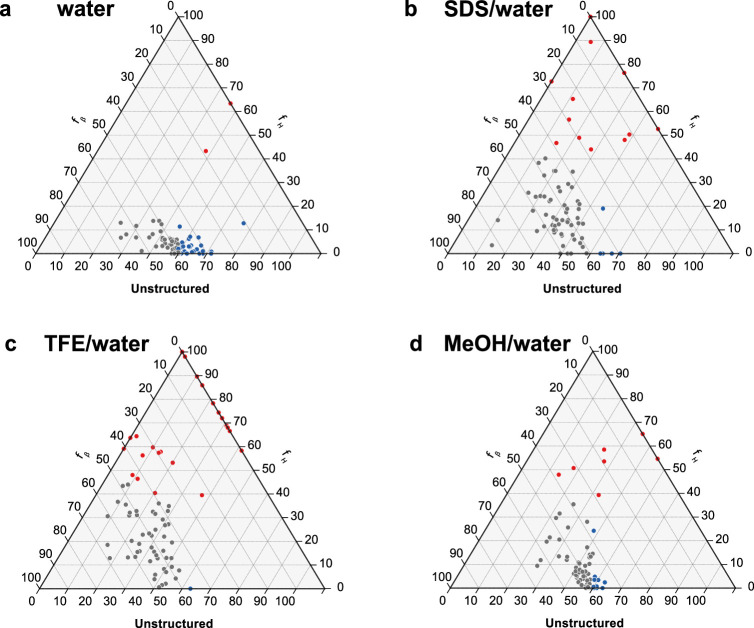
Ternary plots showing the percentage of secondary structure for each peptide (at 50 μmol L^−1^) in four different solvents. **(a)** water, **(b)** Sodium dodecyl sulfate (SDS, 10 mmol L^−1^) in water, **(c)** 60% trifluoroethanol (TFE) in water, and **(d)** 50% methanol (MeOH) in water. Secondary structure fractions were calculated using the BeStSel server. Red dots indicate higher helical fraction, blue dots indicate higher β content, while gray dots represent unstructured peptides. Source data

**Extended Data Fig. 5:**
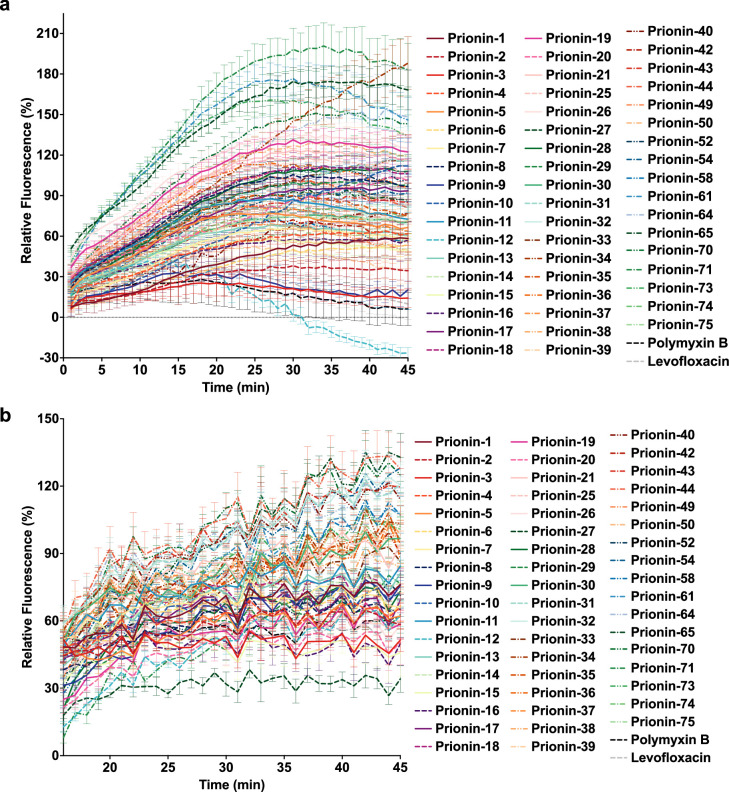
Outer membrane permeabilization and cytoplasmic membrane depolarization of *E. coli* AIC221 induced by prionins. **(a)** Relative fluorescence of all the prionins outer membrane permeabilization, which was assessed using the probe 1-(N-phenylamino)naphthalene (NPN). **(b)** relative fluorescence of all prionins cytoplasmic membrane depolarization. Assays were performed using the hydrophobic probe 3,3′-dipropylthiadicarbocyanine iodide (DiSC_3_-5). Polymyxin B and levofloxacin served as comparison, while buffer, buffer with the probe, and buffer with both probe and bacteria were used as baseline controls for fluorescence. Error bars are the standard deviation obtained from the three replicates and relative fluorescence is shown as a non-linear regression of the fluorescence intensity normalized by the untreated control (buffer). Source data

As membrane disruption is a common mechanism of action for AMPs^1^, we examined whether prionins display similar behaviour. Circular dichroism revealed that many peptides were largely unstructured in water yet adopted more ordered conformations in membrane-mimetic or helix-promoting environments, consistent with inducible folding upon target engagement (Extended Data Figs. 3 and 4). We then evaluated active peptides against *Escherichia coli* AIC221 using *N*-phenyl-1-napthylamine (NPN) uptake and 3,3′-dipropylthiadicarbocyanine iodide (DiSC_3_-5) assays. Many prionins increased outer-membrane permeability and collapsed cytoplasmic-membrane potential, often to levels comparable to or greater than the reference antibiotics included for comparison (Fig. 2a,b and Extended Data Fig. 5). Although the magnitude of each effect varied by peptide, the aggregate pattern supports membrane perturbation as a prevalent mechanism in this class.

A major barrier in AMP development is host toxicity. We therefore assessed haemolysis against human red blood cells (RBCs) and cytotoxicity against human embryonic kidney (HEK293T) cells. Haemolysis was rare: only one of 75 prionins showed haemolytic activity within the tested concentration range. Cytotoxicity was more frequent, indicating that not all prionins are equally developable. Nevertheless, a subset combined antibacterial activity with favourable selectivity, and 16 active peptides showed neither measurable haemolysis nor cytotoxicity at the highest concentrations tested (Fig. 2c). These data indicate that prion-derived antimicrobial activity is not inseparable from mammalian cell toxicity and that tractable leads can be prioritized within the broader library.

**Extended Data Fig. 6:**
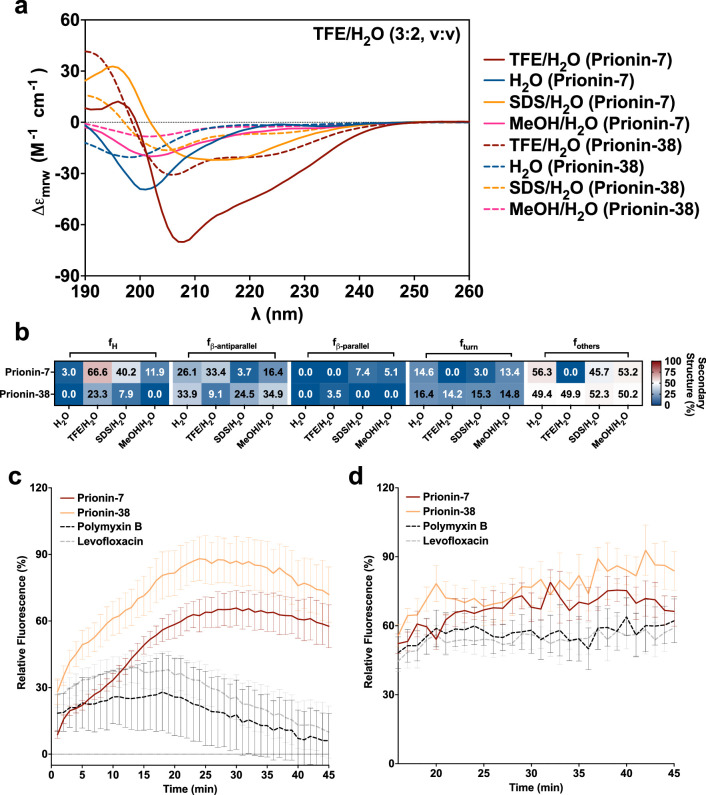
Secondary structure and mechanism of action of prionin-7 and −38. **(a)** Circular dichroism experiments were conducted with prionins-7 and −38, the two peptides tested *in vivo*, using a J-1500 Jasco circular dichroism spectrophotometer. The spectra were recorded in four different media: water, 60% trifluoroethanol in water, 50% methanol in water, and sodium dodecyl sulfate (SDS) in water (10 mmol L^−1^), after three accumulations at 25 °C, using a 1 mm path length quartz cell, between 260 and 190 nm at 50 nm min^−1^, with a bandwidth of 0.5 nm. The concentration of all peptides tested was 50 μmol L^−1^. **(b)** Heatmap with the percentage of secondary structure found for each peptide in the four different solvents. Secondary structure fraction was calculated using the BeStSel server. **(c)** Relative fluorescence of prionins-7 and −38 outer membrane permeabilization, which was assessed using the probe 1-(N-phenylamino)naphthalene (NPN). **(d)** relative fluorescence of prionins-7 and −38 cytoplasmic membrane depolarization. Assays were performed using the hydrophobic probe 3,3′-dipropylthiadicarbocyanine iodide (DiSC_3_-5). Polymyxin B and levofloxacin served as comparison, while buffer, buffer with the probe, and buffer with both probe and bacteria were used as baseline controls for fluorescence. Error bars are the standard deviation obtained from the three replicates and relative fluorescence is shown as a non-linear regression of the fluorescence intensity normalized by the untreated control (buffer). Source data

To determine whether prionins retain activity in vivo, we advanced two lead peptides into a murine skin-abscess model of *A. baumannii* infection (Fig. 2d). Prionin-7, derived from *Thelonectria olida*, and prionin-38, derived from *Caenorhabditis elegans* (Extended Data Fig. 6), were chosen based on low MIC values and favourable toxicity profiles. A single topical dose at 10× MIC administered 1 h after infection significantly reduced bacterial burden relative to untreated controls at day 2, with efficacy comparable to polymyxin B (Fig. 2e). By day 4, prionin-38 maintained an approximately three-log reduction in burden, again tracking the activity of polymyxin B, whereas prionin-7 showed a more modest approximately one-log reduction. No treatment-associated weight loss was observed (Fig. 2f). Thus, prionins predicted from sequence alone can function as anti-infective agents in vivo.

Our findings identify prion and prion-like proteins as an unexpectedly rich reservoir of encrypted AMPs. This expands a growing view that antimicrobial activity can be hidden within proteins not canonically annotated as immune effectors^10,11,16–18^ and extends that concept to prion biology. The results also suggest that the sequence features underlying aggregation-prone proteins can coexist with, or give rise to, membrane-active antimicrobial fragments. At the same time, our study does not show that prionins are naturally released during infection or that they function physiologically as innate immune effectors in their native hosts. Rather, it establishes prion-related proteins as a productive source space for antibiotic discovery and provides a framework for testing whether cryptic peptides contribute to defence in specific biological contexts. We speculate that such antimicrobial molecules may play a role in host immunity, thus potentially linking prion biology and amyloidogenic properties to the immune system.

In summary, deep learning uncovered more than 1,000 candidate prionins from prion-associated proteomes, a subset of which were experimentally active, membrane-perturbing and, in selected cases, effective in vivo. These results connect prion-related sequence space to antimicrobial function and highlight unconventional protein classes as sources of antibiotic leads.

## Methods

### Prion sequence curation and peptide mining

We curated 2,897 UniProt entries annotated with prion keyword (keyword: KW-0640; accessed 24 October 2023), encompassing classical prion proteins as well as prion-related domain–containing proteins. Those included reviewed, unreviewed and isoform entries. Encrypted peptides were defined as substrings of 8–50 amino acid residues derived from these proteins. We were able to obtain 19,324,138 unique candidate encrypted peptides.

### APEX 1.1

Antimicrobial activity was predicted using APEX 1.1, an updated version of our previously published APEX deep learning framework for peptide antibiotic discovery. In brief, peptide sequences were encoded in APEX 1.1 using the same feature-representation strategy described for the original APEX framework. Each peptide was treated as an amino acid sequence with added start and terminal symbols, and each residue was represented by its corresponding AAindex descriptor vector rather than by one-hot encoding. AAindex provides a 566-dimensional representation capturing diverse physicochemical and biochemical properties of amino acids. Unknown or non-amino acid symbols were represented as zero vectors. Sequences were then zero padded to a fixed maximum length (52 tokens including start and terminal symbols) to generate matrix-form inputs for the neural network. APEX 1.1 then uses a peptide-sequence encoder based on recurrent and attention neural network components to generate a learned hidden representation of each peptide sequence, which is then processed by downstream fully connected neural networks for antimicrobial prediction. In our framework, one prediction head was trained on an in-house dataset to perform multitask regression of species-specific MIC values (bacterial strains include *A. baumannii* ATCC 19606*, E. coli* ATCC 11775*, E. coli* AIC221*, E. coli* AIC222*, K. pneumoniae* ATCC 13883, *P. aeruginosa* PAO1*, P. aeruginosa* PA14*, S. aureus* ATCC 12600, methicillin-resistant *S. aureus* (MRSA) ATCC BAA^−^1556, vancomycin-resistant *E. faecalis* ATCC 700802 and vancomycin-resistant *E. faecium* ATCC 700221), whereas a second prediction head was trained on public AMP and non-AMP data to perform AMP/non-AMP classification, thereby improving representation learning through data augmentation. The downstream fully connected networks were implemented as four-layer architectures with layer normalization, rectified linear unit activation and dropout, and ensemble learning was used to improve prediction robustness. Our model was trained on in-house dataset comprising 1,642 peptides and 15,718 MIC measurements across 11 pathogenic strains, together with 19,564 public AMPs and 9,857 non-AMPs. For inactive measurements exceeding the highest tested concentration, MIC values were set to 512 μmol l^−1^. Hyperparameter selection followed the original APEX framework and final predictions were obtained by averaging the selected top-performing ensemble models. Full architectural details, model development and benchmarking have been described previously^10,11^. We used APEX 1.1 to predict the antimicrobial activity of 19,324,138 unique peptide fragments derived from prion proteomes. For each peptide, APEX 1.1 generated predicted MIC values against 11 pathogenic bacterial strains. To obtain an overall measure of predicted antimicrobial potency for ranking, we calculated the median predicted MIC across the 11 strains for each peptide. Peptides with a median predicted MIC of ≤64 μmol l^−1^ were designated as candidate prionins.

### Physicochemical properties analyses

The eight physicochemical properties of peptides, including normalized hydrophobic moment, normalized hydrophobicity, net charge, isoelectric point, disordered conformation propensity, propensity to aggregation in vitro, linear moment and amphiphilicity index, were obtained from the DBAASP server^13^. Note that Eisenberg and Weiss scale^19^ was chosen as the hydrophobicity scale.

### Phylogenetic tree visualization

To obtain the phylogenetic tree, the taxon IDs of 139 organisms containing candidate prionins were uploaded to NCBI Taxonomy Common Tree (ref. ^20^).

### Peptide sequence similarity

We followed the previous practice^10^ to use local alignment to calculate pairwise protein sequence similarity. Let LA(*i, j*) represent the optimal alignment score between protein *i* and protein *j*, a similarity score between this protein pair can be expressed as $\frac{\mathrm{LA}(i,\,j)\,}{\sqrt{\mathrm{LA}\left(i,i\right)\times \mathrm{LA}(j,\,j)\,}}$.

### Peptide sequence space visualization

For (1) predicted antimicrobial prion encrypted peptides, (2) peptides from our in-house dataset and (3) AMPs curated from DBAASP^13^, DRAMP 3.0^21^ and APD3^14^. We calculated pairwise protein sequence similarity and used Uniform Manifold Approximation and Projection (UMAP) to transform the whole sequence similarity matrix into a two-dimensional space. This reduced space is interpretated as a peptide sequence space, allowing us to visualize the how peptides from different sources distribute in it.

### Prionin sequences selection

For 1,179 prionins having ≤64 μmol l^−1^ median MIC by APEX 1.1 prediction, we sorted them by median MIC increasingly and applied the following filtering: (1) the selected peptide should have <70% sequence similarity to all in-house peptides and publicly available AMPs, where the latter ones came from the union of AMPs from DBAASP, APD3 and DRAMP 3.0; and (2) the selected peptides themselves should have <70% sequence similarity. If two peptides break this rule, we keep the more active one. To evaluate the effect of more stringent diversity filters, we also retrospectively applied 50% and 25% sequence-similarity thresholds to the selected candidate set, which would have retained 47 and 0 peptides, respectively.

### Peptide synthesis

All peptides used in the experiments were purchased from AAPPTec and synthesized by solid-phase peptide synthesis using the Fmoc strategy.

### Bacterial strains and growth conditions

In this study, we used the following pathogenic bacterial strains obtained from the American Type Culture Collection (ATCC): *A. baumannii* ATCC 19606, *E. coli* ATCC 11775, *K. pneumoniae* ATCC 13883, *P. aeruginosa* PAO1, *P. aeruginosa* PA14, *S. aureus* ATCC 12600, *S. aureus* ATCC BAA-1556 (methicillin-resistant strain), *E. faecalis* ATCC 700802 (vancomycin-resistant strain) and *E.s faecium* ATCC 700221 (vancomycin-resistant strain). *E. coli* AIC221 (*E. coli* MG1655 phnE_2::FRT (control strain for AIC222)) and *E. coli* AIC222 (*E. coli* MG1655 pmrA53 phnE_2::FRT (polymyxin resistant; colistin-resistant strain)) were kindly donated by Prof. Mark Goulian (University of Pennsylvania). Pseudomonas Isolation (*P. aeruginosa* strains) agar plates were exclusively used in the case of *Pseudomonas* species. All the other pathogens were grown in Luria-Bertani (LB) broth and on LB agar. In all the experiments, bacteria were inoculated from one-isolated colony and grown overnight (16 h) in liquid medium at 37 °C. The following day, inoculums were diluted 1:100 in fresh media and incubated at 37 °C to mid-logarithmic phase.

### RBCs and human embryonic kidney cells

Human embryonic kidney (HEK293T) cells were obtained from the ATCC (CRL-3216). RBCs and human serum were purchased from Zen-Bio. The RBC samples were obtained from the same certified healthy donor (blood type A^−^).

### MIC determination

Broth microdilution assays were performed to determine the MIC values of each peptide. Peptides were added to nontreated polystyrene microtiter 96-well plates and 2-fold serially diluted in sterile water from 1 to 64 μmol L^−1^. Bacterial inoculum at 4 × 10^6^ CFU mL^−1^ in LB medium was mixed 1:1 with the peptide. The MIC was defined as the lowest concentration of peptide able to completely inhibit the bacterial growth after 24 h of incubation at 37 °C. All assays were done in three independent replicates.

### Circular dichroism experiments

The circular dichroism experiments were conducted using a J-1500 circular dichroism spectropolarimeter (Jasco) in the Biological Chemistry Resource Center at the University of Pennsylvania. Experiments were performed at 25 °C, the spectra graphed are an average of three accumulations obtained with a quartz cuvette with an optical path length of 1.0 mm, ranging from 260 to 190 nm at a rate of 50 nm min^−1^ and a bandwidth of 0.5 nm. The concentration of all peptides tested was 50 μmol l^−1^, and the measurements were performed in water, a mixture of trifluoroethanol (TFE) and water in a 3:2 ratio, a mixture of methanol (MeOH) and water in a 1:1 ratio, and sodium dodecyl sulfate (SDS) in water at 10 mmol l^−1^, with respective baselines recorded before measurement. A Fourier transform filter was applied to minimize background effects. Secondary structure fraction values were calculated using the single spectra analysis tool on the server BeStSel^22^.

### Outer-membrane permeabilization assays

The NPN uptake assay was used to evaluate the ability of the peptides to permeabilize the bacterial outer membrane. Inocula of *E. coli* AIC221 were grown to an OD at 600 nm of 0.4, centrifuged (9,391*g* for 3 min), washed and resuspended in 5 mmol l^−1^ HEPES buffer (pH 7.4) containing 5 mmol l^−1^ glucose. The bacterial solution was added to a white 96-well plate (100 μl per well) together with 4 μl of NPN at 0.5 mmol l^−1^. Consequently, peptides diluted in water were added to each well and the fluorescence was measured at *λ*_ex_ = 350 nm and *λ*_em_ = 420 nm over time for 45 min. The relative fluorescence was calculated using the untreated control (buffer + bacteria + fluorescent dye) as baseline and the following equation was applied to reflect the percentage difference between the baselines and the sample:

$$\begin{array}{l}{\rm{Percentage}}\,{\rm{difference}}\\ =\displaystyle \frac{100\times ({{\rm{fluorescence}}}_{{\rm{sample}}}-{{\rm{fluorescence}}}_{{\rm{untreated}}\,{\rm{control}}})}{{{\rm{fluorescence}}}_{{\rm{untreated}}\,{\rm{control}}}}.\end{array}$$

### Cytoplasmic-membrane depolarization assays

The cytoplasmic-membrane depolarization assay was performed using the membrane potential-sensitive dye DiSC_3_-5. *E. coli* AIC221 in the mid-logarithmic phase was washed (9,391*g* for 3 min) and resuspended at 0.05 OD ml^−1^ (optical value at 600 nm) in HEPES buffer (pH 7.2) containing 20 mmol l^−1^ glucose and 0.1 mol l^−1^ KCl. DiSC_3_-5 at 20 μmol l^−1^ was added to the bacterial suspension (100 μl per well) for 15 min to stabilize the fluorescence, which indicates the incorporation of the dye into the bacterial membrane, and then the peptides were mixed 1:1 with the bacteria to a final concentration corresponding to their MIC values. Membrane depolarization was then followed by reading changes in the fluorescence (*λ*_ex_ = 622 nm and *λ*_em_ = 670 nm) over time for 60 min. The relative fluorescence was calculated using the untreated control (buffer + bacteria + fluorescent dye) as baseline and the following equation was applied to reflect the percentage difference between the baselines and the sample:

$$\begin{array}{l}{\rm{Percentage}}\,{\rm{difference}}\\ =\displaystyle \frac{100\times ({{\rm{fluorescence}}}_{{\rm{sample}}}-{{\rm{fluorescence}}}_{{\rm{untreated}}\,{\rm{control}}})}{{{\rm{fluorescence}}}_{{\rm{untreated}}\,{\rm{control}}}}.\end{array}\,\,$$

### Haemolytic activity assays

To evaluate the release of haemoglobin from human erythrocytes upon treatment of each of the encrypted peptides, human RBCs were obtained from Zen-Bio (male donor, blood type A^−^) obtained from heparin anticoagulated blood. RBCs were washed with PBS (pH 7.4) four times by centrifugation at 800*g* for 10 min. Aliquots of 200-fold diluted cells (75 μl) were mixed with peptide solution (0.78–100 μmol l^−1^; 75 μl), and the mixture was incubated for 4 h at room temperature. After the incubation, the plate was centrifuged at 1,300*g* for 10 min to precipitate cells and debris, and 100 μl of supernatant from each well were transferred to a new 96-well plate for absorbance reading (405 nm) using an automatic plate reader. The percentage of haemolysis was defined by comparison with negative control (samples containing PBS) and positive control (samples containing 1% (v/v) SDS in PBS solution).

$$\begin{array}{l}{\rm{Haemolysis}}({\rm{ \% }})\\ =\displaystyle \frac{100\times ({{\rm{Absorbance}}}_{405{\rm{nm}}\,{\rm{peptide}}}-{{\rm{Absorbance}}}_{405{\rm{nm}}\,{\rm{negative}}\,{\rm{control}}})}{({{\rm{Absorbance}}}_{405{\rm{nm}}\,{\rm{positive}}\,{\rm{control}}}-{{\rm{Absorbance}}}_{405{\rm{nm}}\,{\rm{negative}}\,{\rm{control}}})}.\end{array}$$

### Cytotoxicity assays

The cells were cultured in high-glucose Dulbecco’s modified Eagle’s medium supplemented with 1% penicillin and streptomycin (antibiotics) and 10% fetal bovine serum and grown at 37 °C in a humidified atmosphere containing 5% CO_2_.

One day before the experiment, 100 μl aliquots of human embryonic kidney (HEK293T) cells, at a concentration of 50,000 cells per ml, were seeded into each well of 96-well plates (5,000 cells per well). Following cell attachment, the HEK293T cells were treated with increasing concentrations of peptides (ranging from 8 to 128 μmol l^−1^) and incubated for 24 h. After the exposure period, cytotoxicity was assessed using the 3-(4,5-dimethylthiazol-2-yl)-2,5-diphenyltetrazolium bromide (MTT) assay. Specifically, the MTT reagent was prepared at a concentration of 0.5 mg ml^−1^ in phenol red-free medium and used to replace the peptide-containing supernatants (100 μl per well). The plates were then incubated for 4 h at 37 °C in a humidified atmosphere with 5% CO_2_, facilitating the formation of insoluble formazan crystals. These crystals were subsequently dissolved in 0.04 mol l^−1^ hydrochloric acid prepared in anhydrous isopropanol. Absorbance was measured at 570 nm using a spectrophotometer to quantify cell viability. All experiments were conducted in triplicate (three biological replicates).

### Skin abscess infection mouse model

The back of 6-week-old female CD-1 mice under anaesthesia were shaved and injured with a superficial linear skin abrasion made with a needle. An aliquot of *A. baumannii* ATCC 19606 (8.33 × 10^5^ c.f.u. ml^−1^; 20 μl) previously grown in LB medium until 0.5 OD ml^−1^ (optical value at 600 nm) and then washed twice with sterile PBS (pH 7.4, 9,391*g* for 3 min) added to the scratched area. Peptides diluted in sterile water at their MIC value were administered to the wounded area 1 h postinfection. At 2 and 4 days postinfection, animals were euthanized and a uniform excision of the scarified skin was excised, homogenized using a bead beater (25 Hz for 20 min), tenfold serially diluted and plated on McConkey agar plates for c.f.u. quantification. The experiments were performed using six mice per group. Mice were single housed to avoid cross-contamination and maintained under a 12-h light/dark cycle at 22 °C with humidity controlled at 50%. The skin abscess infection mouse model was revised and approved by the University Laboratory Animal Resources from the University of Pennsylvania (protocol no. 806763).

### Quantification and statistical analysis

#### Reproducibility of the experimental assays

All assays were performed in three independent biological replicates as indicated in each figure legend and in the relevant Methods sections. The values obtained for haemolytic and cytotoxic activity were estimated by nonlinear regression based on the screen of peptides in a gradient of concentrations and represent the haemolytic and cytotoxic concentration values needed to lyse and kill 50% of the cells present in the experiment. In the skin abscess mouse model, we used six mice per group following established protocols approved by the University Laboratory of Animal Resources of the University of Pennsylvania.

#### Statistical tests

In the mouse experiments, all the raw data were log_10_ transformed and the statistical significance was determined using one-way analysis of variance (ANOVA) followed by Dunnett’s test. All the *P* values are shown for each of the groups, and all groups were compared with the untreated control group.

#### Statistical analysis

All calculations and statistical analyses of the experimental data were conducted using GraphPad Prism v.11. Statistical significance between different groups was calculated using the tests indicated in each figure legend. No statistical methods were used to predetermine sample size.

### Reporting summary

Further information on research design is available in the Nature Portfolio Reporting Summary linked to this article.

## Supplementary information

Reporting Summary

## Source data

Source Data Fig. 1 UMAP visualization of sequence space comparing candidate prionins with peptides from the in-house APEX dataset and curated AMP databases (**c**). Heat map of MIC values for the 75 synthesized prionins tested against 11 clinically relevant pathogens, including resistant strains (**d**).

Source Data Fig. 2 Outer-membrane permeabilization of *E. coli* AIC221 measured by NPN uptake for representative active prionins tested at their MIC values (**a**). Cytoplasmic-membrane depolarization measured by DiSC_3_-5 for representative active prionins. Initial time reported is 15 min, after the stabilization of the probe in the membrane, when the peptide was added (**b**). Comparison of antimicrobial activity with haemolytic (HC_50_) and cytotoxic (CC_50_) measurements identifies a subset of selective prionins (**c**). Bacterial burden in a murine skin-abscess model of *A. baumannii* infection following topical treatment with prionin-7, prionin-38 or polymyxin B (**e**). Mouse body weight over the course of treatment (**f**).

Source Data Extended Data Fig. 1 Bars show peptide counts per phylum.

Source Data Extended Data Fig. 2 Comparison of amino acid frequency in prionins with known AMPs from the DBAASP, APD3, and DRAMP 3.0 databases (**a**). Distribution of some of the most important physicochemical properties for peptides with predicted antimicrobial activity, compared with AMPs from DBAASP, APD3, and DRAMP 3.0: net charge(**b**), length (**c**) and normalized hydrophobicity (**d**). Isoelectric point (**e**), normalized hydrophobic moment (**f**) and linear moment (**g**), reflecting the amphipathicity of the molecules, which directly influences their interactions with bacterial membranes. Amphiphilicity index (**h**) and disordered conformation propensity (**i**), both of which are closely correlated with the mechanism of action, specifically how peptides interact with membrane lipids to exert antimicrobial activity. Propensity to aggregate in vitro, correlated with the supramolecular arrangement of the molecules and potential toxicity(**j**).

Source Data Extended Data Fig. 3 Circular dichroism spectra in four different media: water, 60% TFE in water, 50% methanol in water and SDS in water (10 mmol l^−1^) (**a**). Heat map with the percentage of secondary structure found for each peptide in the four different solvents (**b**).

Source Data Extended Data Fig. 4 Water (**a**), SDS (10 mmol l^−1^) in water (**b**), 60% TFE in water (**c**) and 50% methanol (MeOH) (**d**) in water.

Source Data Extended Data Fig. 5 Relative fluorescence of all the prionins outer membrane permeabilization, which was assessed using the probe NPN (**a**). Relative fluorescence of all prionins cytoplasmic membrane depolarization (**b**). Assays were performed using the hydrophobic probe DiSC_3_-5.

Source Data Extended Data Fig. 6 Circular dichroism experiments with prionins-7 and −38, the two peptides tested in vivo (**a**). The spectra were recorded in four different media: water, 60% trifluoroethanol in water, 50% methanol in water and SDS in water (10 mmol l^−1^). Heat map with the percentage of secondary structure found for each peptide in the four different solvents (**b**). Relative fluorescence of prionins-7 and -38 outer-membrane permeabilization, which was assessed using the probe NPN (**c**). Relative fluorescence of prionins-7 and -38 cytoplasmic-membrane depolarization. Assays were performed using the hydrophobic probe DiSC_3_-5 (**d**).

## Data Availability

This study did not generate new unique reagents. All reviewed classical prion proteins as well as prion-related domain–containing proteins sequences (keyword: KW-0640) can be downloaded from UniProt (https://www.uniprot.org/). The AMPs analysed in this study were obtained from publicly available databases, including DBAASP (https://dbaasp.org/home), APD3 (https://aps.unmc.edu/) and DRAMP (http://dramp.cpu-bioinfor.org/). Further information and requests for resources should be directed to the corresponding author. Source data are provided with this paper.

## Code availability

APEX 1.1 is available via GitLab at https://gitlab.com/machine-biology-group-public/apex-pathogen.
